# Targeted Deletion of the *USTA* and *UvSLT2* Genes Efficiently in *Ustilaginoidea virens* With the CRISPR-Cas9 System

**DOI:** 10.3389/fpls.2018.00699

**Published:** 2018-05-24

**Authors:** Yafeng Liang, Yu Han, Chenfang Wang, Cong Jiang, Jin-Rong Xu

**Affiliations:** ^1^State Key Laboratory of Crop Stress Biology for Arid Areas, Purdue-NWAFU Joint Research Center, College of Plant Protection, Northwest A&F University, Yangling, China; ^2^Department of Botany and Plant Pathology, Purdue University, West Lafayette, IN, United States

**Keywords:** rice false smut, MAP kinase, pathogenesis, gene knockout, ustiloxins

## Abstract

*Ustilaginoidea virens* is the causal agent of rice false smut, one of the major fungal diseases of rice. However, there are only limited molecular studies with this important pathogen due to the lack of efficient approaches for generating targeted gene disruption mutants. In this study, we used the CRISPR-Cas9 system to efficiently generate mutants deleted of the *USTA* ustiloxin and *UvSLT2* MAP kinase genes. Three gRNA spacers of *USTA*, UA01, UA13, and UA21, were expressed with the RNAP III promoter of Gln-tRNA. For all of them, the homologous gene replacement frequency was higher when the Cas9 and gRNA constructs were transformed into *U. virens* on the same vector than sequentially. UA01, the spacer with the highest on-target score, had the highest knockout frequency of 90%, which was over 200 times higher than that of *Agrobacterium tumefaciens*-mediated transformation (ATMT) for generating *ustA* mutants. None of these *USTA* spacers had predicted off-targets with 1 or 2-nt variations. For predicted off-targets with 3 or 4-nt variations, mutations were not detected in 10 *ustA* mutants generated with spacer UA13 or UA21, indicating a relatively low frequency of off-target mutations in *U. virens*. For *UvSLT2*, the homologous gene replacement frequency was 50% with CRISPR-Cas9, which also was significantly higher than that of ATMT. Whereas *ustA* mutants had no detectable phenotypes, *Uvslt2* mutants were slightly reduced in growth rate and reduced over 70% in conidiation. Deletion of *UvSLT2* also increased sensitivity to cell wall stresses but tolerance to hyperosmotic or oxidative stresses. Taken together, our results showed that the CRISPR-Cas9 system can be used as an efficient gene replacement or editing approach in *U. virens* and the UvSlt2 MAP kinase pathway has a conserved role in cell wall integrity.

## Introduction

Rice false smut is a destructive disease caused by the ascomycete *Ustilaginoidea virens* (Teleomorph *Villosiclava virens*). In the past decade, this disease has becoming one of the major fungal diseases that threaten rice production (Zhou et al., 2008). In infected kernels, smut balls containing darkly-pigmented chlamydospores are developed instead of rice seeds. *U. virens* is also a producer of toxic secondary metabolites, including ustiloxins that are toxic to plants and animals by interfering with the microtubule functions (Tsukui et al., 2015).

Unlike many other plant pathogenic fungi, there are only limited molecular genetic studies in *U. virens* although its genome was sequenced and published in Zhang et al. (2014). To date, only the *UvHOG1, UvSUN2, Uvt3277*, and *UvPRO1* genes have been functionally characterized by deletion or disruption in this important plant pathogenic fungus (Yu et al., 2015; Lv et al., 2016; Zheng et al., 2016, 2017). One major bottleneck for molecular genetic studies with *U. virens* is its low homologous recombination frequency using the conventional gene replacement approaches. Mutants disrupted of the *UvSUN2, Uvt3277*, and *UvPRO1* genes were generated by random insertional mutagenesis instead of targeted gene deletion or disruption (Yu et al., 2015; Lv et al., 2016; Zheng et al., 2017). For *UvHOG1*, the only *U. virens* gene with mutants generated by targeted gene deletion, less than 0.5% of hygromycin-resistant transformants generated by *Agrobacterium tumefaciens*-mediated transformation (ATMT) were confirmed to true deletion mutants (Zheng et al., 2016). The homologous gene replacement frequency is about 10 times higher in many other plant pathogenic fungi such as the rice blast fungus *Magnaporthe oryzae* and wheat scab fungus *Fusarium graminearum*, in which 100s of pathogenicity factors having been characterized (Wang et al., 2011; Choi et al., 2013).

The clustered regularly interspaced short palindromic repeats (CRISPR)-associated RNA-guided DNA endonuclease Cas9 has been extensively used for gene editing in plants and animals by taking advantage of the simple design of a single crRNA:tracrRNA chimeric guide RNA (gRNA). The gRNA contains a 20-bp target sequence that can guide Cas9 to the target locus and cause double-strand breaks (DSB) by Cas9, which triggers targeted gene editing or replacement by homologous recombination (HR) in organisms that have no or very low HR frequency (Cong et al., 2013). Although most filamentous fungi have a higher homologous recombination frequency than animals and plants, the CRISPR-Cas9 system has been reported to improve the HR frequency for targeted gene deletion in several ascomycetes, including *Trichoderma reesei, M. oryzae, Neurospora crassa, Alternaria alternata, Penicillium chrysogenum*, and *Aspergillus niger* (Arazoe et al., 2015; Liu et al., 2015; Matsu-ura et al., 2015; Kuivanen et al., 2016; Pohl et al., 2016; Wenderoth et al., 2017).

In this study, we used the CRISPR-Cas9 system to functionally characterize the *USTA* ustiloxin and *UvSLT2* MAP kinase genes in *U. virens*. Because the U6 promoter is not conserved in *U. virens*, the promoter of its Gln-tRNA gene was used as the RNAP III promoter to express the sgRNA constructs. For both *USTA* and *UvSLT2*, the homologous gene replacement frequency was significantly higher with the CRISPR-Cas9 system than the conventional ATMT approach. For the three *USTA* gRNA spacers tested, the gene replacement frequency was higher when the Cas9 and gRNA constructs were transformed into *U. virens* on the same vector than sequentially. The gRNA spacer with the high on-target score had the highest HR frequency and none of the *ustA* mutants assayed had mutations at predicted off-targets, suggesting a low frequency of off-target mutations. Whereas *ustA* mutants had no detectable phenotypes in cultures, *Uvslt2* mutants were reduced in growth rate and conidiation and had increased sensitivity to cell wall stresses. Taken together, our results showed that the CRISPR-Cas9 system can be used as an efficient gene replacement or editing approach in *U. virens* and the UvSlt2 MAP kinase pathway has a conserved role in cell wall integrity.

## Results

### Construction of the tRNA-gRNA Vectors for *U. virens* Transformation

**FIGURE 1:**
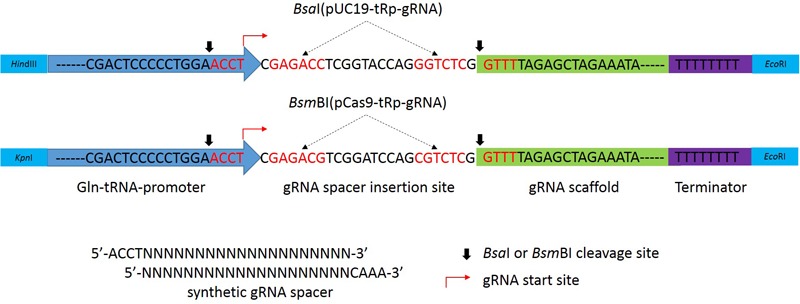
Diagram of the modified Gln-tRNA-gRNA cassettes in pUC19-tRp-gRNA and pCas9-tRp-gRNA vectors. The blue arrow on the left is the Gln-tRNA promoter that is followed by the gRNA spacer insert site, gRNA scaffold (green), and RNA polyIII terminator (purple) sequences. The gRNA spacers are synthesized by annealing the sense and antisense oligonucleotides with 5′-ACCT and 3′-CAAA overhangs and inserted into the gRNA spacer insertion site of *Bsa*I-digested pUC19-tRp-gRNA (upper) or *Bsm*BI-digested pCas9-tRp-gRNA (lower). The gRNA start site is marked with a red arrow. The cleavage sites of type II restriction enzyme *Bsa*I and *Bsm*BI are marked with black arrows.

To develop gRNA vectors suitable for the rice false smut fungus, we first identified the glutaminyl-tRNA (Gln-tRNA) gene in the *U. virens* genome (Zheng et al., 2014; Mefferd et al., 2015; Xie et al., 2015) with the tRNAscan-SE program (Lowe and Eddy, 1997). The 72-bp Gln-tRNA region was synthesized and fused with the 108-bp gRNA by fusion PCR with primers listed in Supplementary Table S1. The resulting PCR product with two *Bsa*I sites between Gln-tRNA and gRNA was then cloned between the *Hin*dIII and *Eco*RI sites of plasmid pUC-H1-gRNA that carries the geneticin-resistance marker (Gen^R^) (Zheng et al., 2014) to generate the pUC19-tRp-gRNA vector (**Figure 1**). A short cassette with two *Bsm*BI sites was generated by annealing the sense and antisense oligonucleotides LINK-F and LINK-R (Supplementary Table S1) and inserted between the two *Bsa*I sites of pUC19-tRp-gRNA by Golden Gate cloning (Ran et al., 2013; Arazoe et al., 2015). In the resulting construct, the two *Bsa*I sites in the Gln-tRNA-gRNA cassette were replaced with two *Bsm*BI sites. The modified Gln-tRNA-gRNA cassette was then inserted between the *Kpn*I and *Eco*RI sites of pCRISPR/Cas-U6-1 (Arazoe et al., 2015) to generate the pCas9-tRp-gRNA vector (**Figure 1**).

### Generation of *U. virens* Transformants Expressing the Cas9 Enzyme

To generate *U. virens* transformants expressing the Cas9 enzyme alone, the pDHt/sk-PC Cas9 vector (Liu et al., 2015) carrying the hygromycin phosphotransferase (*hph*) cassette was transformed into the wild-type strain P1 (Zheng et al., 2017) by *A. tumefaciens*-mediated transformation (ATMT). Transformants resistant to hygromycin were isolated and analyzed by PCR. Strain CS-2 (**Table 1**) was one of the transformants that were confirmed to contain the transforming Cas9 vector.

**Table 1:** Strains and vectors used in this study.

Strain	Brief description	Reference
P1	Wild-type	Zheng et al., 2017
CS-2	Transformant of P1 expressing the pDHt/sk-PC	This study
MS-1	*Uvslt2* deletion mutant of P1	This study
MS-2	*Uvslt2* deletion mutant of P1	This study
MS-4	*Uvslt2* deletion mutant of P1	This study
MS-5	*Uvslt2* deletion mutant of P1	This study
MS-8	*Uvslt2* deletion mutant of P1	This study
MS-9	*Uvslt2* deletion mutant of P1	This study
MS-11	*Uvslt2* deletion mutant of P1	This study
MS-12	*Uvslt2* deletion mutant of P1	This study
MS-15	*Uvslt2* deletion mutant of P1	This study
MU-45	*ustA* deletion mutant of P1	This study
MU-47	*ustA* deletion mutant of P1	This study
MU-49	*ustA* deletion mutant of P1	This study
MU-52	*ustA* deletion mutant of P1	This study
MU-54	*ustA* deletion mutant of P1	This study
MU-60	*ustA* deletion mutant of P1	This study
**Vectors**		
pUC-H1-gRNA	Vector with the H1 promoter for gRNA expression	Zheng et al., 2014
pDHt/sk-PC	pDH1/tk-P_pdc_-toCas9-T_pdc_	Liu et al., 2015
pCRISPR/Cas-U6-1	Cas9-gRNA vector with the U6 promoter	Arazoe et al., 2015
pCBDW	*Agrobacterium* binary vector	Zheng et al., 2016
pUC19-tRp-gRNA	Gln-tRNA promoter of gRNA	This study
pCAS9-tRp-gRNA	Cas9-gRNA vector with the tRNA promoter	This study
pCas9-tRp-UA01	Cas9-gRNA vector with the UA01 spacer	This study
pCas9-tRp-UA13	Cas9-gRNA vector with the UA13 spacer	This study
pCas9-tRp-UA21	Cas9-gRNA vector with the UA21 spacer	This study
pCas9-tRp-SLT01	Cas9-gRNA vector with the SLT01 spacer	This study
pHY2016A	pCAS9: tRp-SLT01 gRNA	This study

### The Gene Replacement Frequency for *USTA* Is Over 60% With the CRISPR-Cas9 System

**FIGURE 2:**
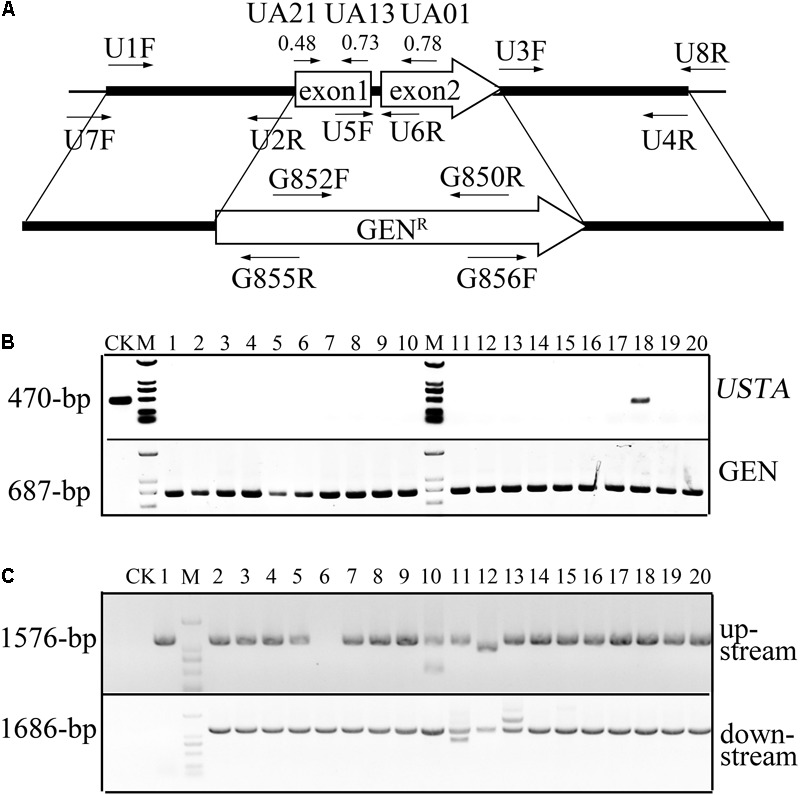
The *USTA* gene and deletion mutants. **(A)** The *USTA* gene and gRNA spacers. The position and direction of gRNA spacers and primers used to generate and screen *ustA* deletion mutants are marked with arrows. The on-target scores of spacers UA01, UA13, and UA21 are labeled in the bracket. **(B)** PCR assays for the deletion of *USTA* (upper panel) and presence of the geneticin-resistance gene (lower panel) in 20 transformants generated with pCas9-tRp-gRNA-UA01 (spacer UA01). The 470-bp *USTA* fragment was only amplified in the wild-type (CK) and transformant 18. M: 1-kb DNA ladder marker. **(C)** PCR assays to verify gene replacement events in 20 putative *ustA* deletion mutants. The 1576-bp upstream and 1686-bp downstream recombination products were amplified with primer pairs USTA7F(U7F)/G855R and USTA8R(U8R)/G856F, respectively. Amplification with P1 was used as the negative control. M: 1-kb DNA ladder marker.

To test the efficiency of CRISPR-Cas9 for generating knockout mutants in *U. ravens*, we first identified the predicted gene UV8b_7487 as the ortholog of *ustA* of *Aspergillus flavus* (Tsukui et al., 2015) and named it *USTA* in this study. In *A. flavus, ustA* encodes an oligopeptide that is processed post-translationally to ustiloxins (Tsukui et al., 2015). Three gRNA spacers, UA01, UA13, and UA21 (Supplementary Table S1), were designed with the sgRNA designer program for best on-target scores (Doench et al., 2014, 2016). Among them, UA01 had the highest predicted on-target score (**Figure 2A**). All of these three spacers were cloned into the *Bsa*I-digested pUC19-tRp-gRNA vector by Golden Gate Cloning (Arazoe et al., 2015). The resulting constructs were verified by sequencing analysis and transformed into protoplasts of the Cas9-expressing transformant CS-2 (**Table 1**). Transformants resistant to geneticin G418 were isolated and screened by PCR with primers USTAF5 and USTA6R (Supplementary Table S1) for deletion of *USTA* (**Figure 2B**), and further verified by PCR with primer pairs USTA7F/G855R and USTA8R/G856F for homologous recombination in both upstream and downstream flanking sequences (**Figure 2C**).

For spacer UA01, 27 of the 36 G418-resistant transformants were confirmed to be *ustA* deletion mutants, suggesting that the homologous gene replacement frequency was as high as 75%. For spacers UA13 and UA21, 21 *ustA* deletion mutants each were identified by PCR after screening 34 and 36 G418-resistant transformants, respectively. Five of these *ustA* deletion mutants identified by PCR were selected for further verification by Southern blot analysis. All of them were confirmed to be deleted of the *USTA* gene (Supplementary Figure S1). The *ustA* mutants were normal in growth, colony morphology, and conidiation and had no defects in response to different stresses. These results indicated that the homologous gene replacement frequency varied among different gRNA spacers from 60 to 75% in *U. virens*. Space UA01 with the highest on-target score had the highest gene replacement frequency.

For comparison, we also generated *ustA* deletion mutants by the conventional ATMT approach (Zheng et al., 2016). The *USTA* gene replacement construct was generated by overlapping PCR and cloned into the *Agrobacterium* vector pCBDW-HPH (Zheng et al., 2016), which was then transformed into the wild-type strain P1. After screening over 600 ATMT transformants, only one *ustA* deletion mutant was identified, indicating a homologous replacement frequency of <0.2%. Therefore, the CRISPR-Cas9 system significantly increases the homologous gene replacement frequency in *U. virens* in comparison with ATMT.

### Transformation of the Cas9 and gRNA Constructs on the Same Vector Further Increases the Gene Replacement Frequency in *U. virens*

Three gRNA spacers of *USTA*, UA01, UA13, and UA21, also were cloned into the *Bsm*BI-digested pCas9: tRp-gRNA vector by Golden Gate Cloning (Arazoe et al., 2015). All the resulting constructs were verified by sequencing analysis and transformed into protoplasts of the wild-type strain P1. For spacer UA01, 27 of the 30 G418-resistant transformants were *ustA* deletion mutants. For spacers UA13 and UA21, 25, and 26 *ustA* deletion mutants were identified after screening 29 and 30 G418-resistant transformants, respectively. Therefore, the homologous gene replacement frequency was 90, 86, and 87%, respectively, for spacers UA01, UA13, and UA21, which was higher than transforming these gRNA spacers into the Cas9-expressing transformant CS-2. These results indicate that transformation of the Cas9 and gRNA constructs into *U. virens* on the same vector further increased the homologous gene replacement frequency.

### No Off-Target Mutations Are Detected in *ustA* Mutants Generated With CRISPR-Cas9

**FIGURE 3:**
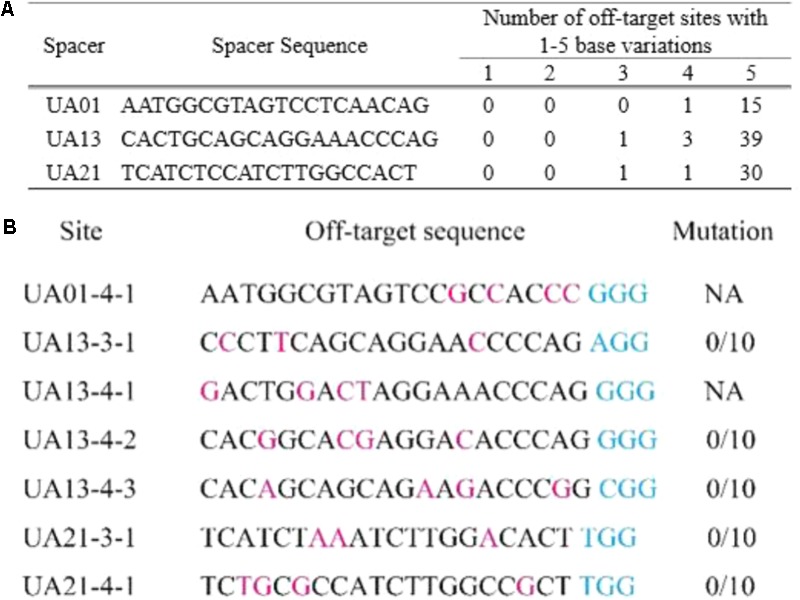
The predicted off-targets of three *USTA* gRNA spacers. **(A)** The sequences of three *USTA* gRNA spacers and numbers of their off-targets with 1–5 nucleotide differences. The off-target sites of gRNA spacers UA01, UA13, and UA21 were predicted with the Cas9off program. All of them have no off-targets with only one or two nucleotide variations. Spacer UA01 has only one off-target with four nucleotide variations. **(B)** Sequences of the off-targets of with fewer than five nucleotide variations from *USTA* gRNA spacers and experimental verification results. Nucleotides in the off-targets differing from the gRNA sequences are in pink. The PAM sequences (NGG) are in blue. For each gRNA spacer, the off-target sites were amplified and sequenced from 10 corresponding *ustA* deletion mutants. No mutations were detected in any of the 10 *ustA* mutants (0/10) at UA13-3-1, UA13-4-2, UA13-4-3, UA21-3-1, and UA21-4-1. NA, no data available due to PCR failures.

For the three gRNA spacers of *USTA*, potential off-targets of the RNA-guided nuclease (RNG) Cas9 were analyzed with the Cas9off program (Guo et al., 2014). None of them had off-targets with less than two nucleotide variations, indicating that they are highly specific in the *U. virens* genome. Spacers UA01, UA13, and UA21 have 1, 4, and 2 off-targets with four or less nucleotide variations, respectively (**Figure 3A**).

We then selected the *ustA* mutants generated with the pCas9: tRp-gRNA constructs (Cas9 + gRNA spacers) for experimental verification. For each spacer, 10 *ustA* mutants were selected for PCR and sequencing analysis for possible off-targets with 3 or 4 mismatches (**Figure 3A**). For spacer UA13, no mutations were detected at the predicted off-targets UA13-3-1, UA13-4-2, and UA13-4-3 in any of the 10 *ustA* mutants sequenced (**Figure 3B**). Similar results were obtained with transformants generated with spacer UA21. None of the 10 *ustA* mutants had mutations at the off-targets UA21-3-1 and UA21-4-1 (**Figure 3B**). These results suggested that mutations at the off-targets of these gRNA spacers likely occur at a relatively low frequency in *U. viren*, which may be related to the low complexity of *U. virens* genome and high specificity of gRNA spacers.

### The CRISPR-Cas9 System Also Increases the Knockout Efficiency for the *UvSLT2* MAPK Gene

**FIGURE 4:**
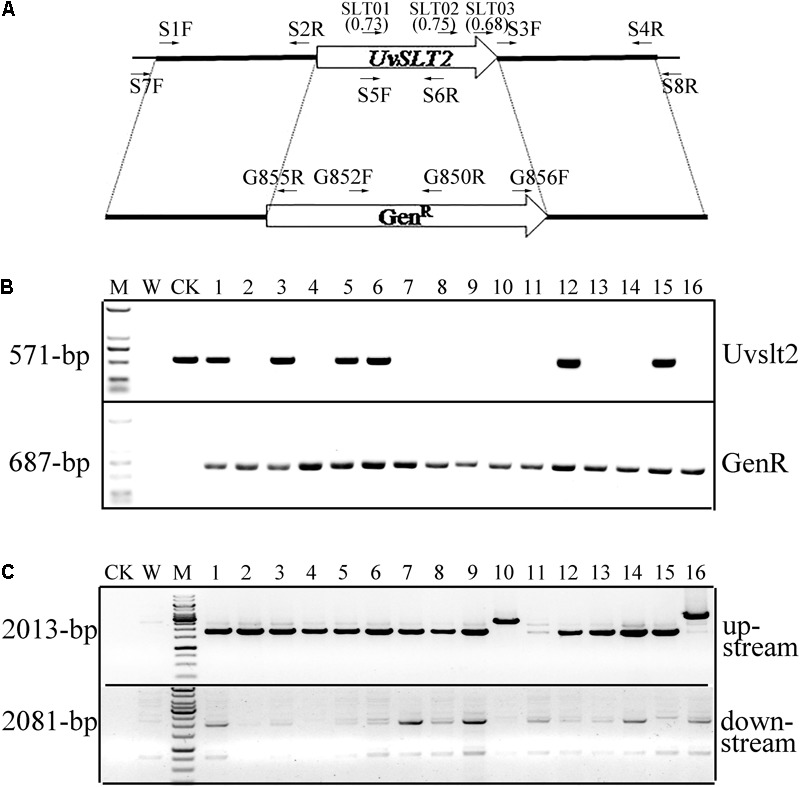
The *UvSTL2* gene and deletion mutants. **(A)** The *UvSLT2* gene and gRNA spacers. The position and direction of gRNA spacers and primers used to generate and screen *Uvslt2* deletion mutants are marked with arrows. The on-target scores of spacers SLT01, SLT02, and SLT03 are labeled in the bracket. **(B)** PCR assays for the deletion of *UvSTL2* (upper panel) and presence of the geneticin-resistance gene (lower panel) in 16 transformants generated with pCas9-tRp-gRNA-SLT01 (spacer SLT01). The 571-bp *UvSTL2* fragment was only amplified in the wild-type (CK) and transformant 1, 3, 5, 6, 12, and 15. M: 1-kb DNA ladder marker. **(C)** PCR assays to verify gene replacement events in 16 putative *Uvslt2* deletion mutants. The 2013-bp upstream and 2081-bp downstream recombination products were amplified with primer pairs SLT27F(S7F)/G855R and SLT8R(S8R)/G856F, respectively. Amplification with P1 was used as the negative control. M: 1-kb DNA ladder marker.

To further test the gene knockout efficiency of the CRISPR-Cas9 system in *U. virens*, we then selected the *UvSLT2* MAP kinase (MAPK) gene that is orthologous to yeast *SLT2* (Gustin and Albertyn, 1998; Xu et al., 1998). Three *UvSLT2* gRNA spacers were designed (Supplementary Table S1) with the gRNA designer program (Doench et al., 2014, 2016). SLT02 had the highest predicted on-target score of 0.75 (**Figure 4A**) and was the only gRNA spacer used to for *UvSLT2* deletion. Spacer SLT02 was cloned into the *Bsm*BI-digested pCas9-tRp-gRNA vector by Golden Gate Cloning. The resulting construct pHY2016A was verified by sequencing analysis and transformed into protoplasts of the wild-type strain P-1. Among the 50 G418-resistant transformants analyzed, 25 of them were confirmed to be *Uvslt2* deletion mutants by PCR screening (**Figure 4B**), and verified for homologous recombination in both flanking sequences (**Figure 4C**), indicating a homologous gene replacement frequency of 50%. Therefore, the homologous gene replacement frequency of the CRISPR-Cas9 system varied among different spacers and different genes in *U. virens*.

For comparison, we also generated the *UvSLT2* gene replacement construct and transformed it into the wild-type strain UV-8b by ATMT. After screening 238 hygromycin-resistant transformants, two *Uvslt2* deletion mutants were identified by PCR analysis. Therefore, the homologous gene replacement frequency was approximately 0.8% for *UvSLT2* with the ATMT approach, which was significantly lower than that of the CRISPR-Cas9 system.

### The *Uvslt2* Mutant Has Increased Sensitivity to Cell Wall and Cytoplasm Membrane Stresses

**FIGURE 5:**
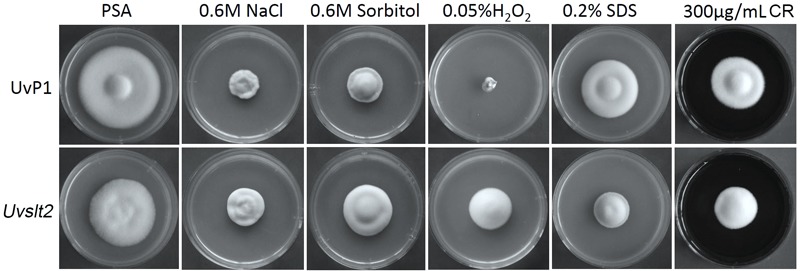
Defects of the *Uvslt2* mutant in response to different stresses. The wild-type strain P1 and *Uvslt2* mutant were cultured on regular PSA plates or PSA with 0.6 NaCl, 0.6 M sorbitol, 0.05% H_2_O_2_, 0.2% SDS, or 300 μg/ml Congo red (CR). Typical cultures were photographed after incubation for 14 days.

The MAP kinases orthologous to Slt2 are known to regulate cell wall integrity and other differentiation processes in fungal pathogens (Hamel et al., 2012). The *Uvslt2* mutant was slightly reduced in growth rate (**Table 2**) and produced colonies with shorter aerial hyphae on PSA and 5xYEG plates in comparison with the wild-type (**Figure 5**). Conidiation was reduced over 70% in the *Uvslt2* mutant (**Table 2**). In the presence of 300 mg/L Congo red or 0.2% SDS, the *Uvslt2* mutant was significantly more reduced in growth rate than the wild-type on PSA plates (**Figure 5** and **Table 3**), indicating that deletion of *UvSLT2* increased sensitivities to cell wall and cytoplasm membrane stresses. However, the *Uvslt2* mutant grew faster than the wild-type on PSA plates with 0.6 M NaCl, 0.6 M Sorbitol, or 0.05% H_2_O_2_ (**Figure 5** and **Table 3**). It appeared that deletion of *UvSLT2* increased tolerance to hyperosmotic and oxidative stresses.

**Table 2:** Defects of the *Uvslt2* mutant in growth and conidiation.

Strain	Growth rate (mm/d)^a^		Conidiation^b^ (10^6^ conidia/ml)
	PDA	5xYEG	
P1 (wild-type)	2.88 ± 0.04^A^	2.86 ± 0.02^A^	3.0 ± 0.5^A^
MS-2 (*Uvslt2*)	2.31 ± 0.13^B^	2.23 ± 0.28^B^	0.9 ± 0.1^B^

*^*a*^The average growth rate and standard deviation (mean ± SD) were calculated from at least three independent measurements. ^*b*^Conidiation in 6-day-old PSB cultures. Data from three replicates were analyzed with the protected Fisher’s least significant difference (LSD) test. Different letters mark statistically significant differences (*P* ≤ 0.05).*

**Table 3:** Defects of the Uvslt2 mutant in response to different stresses.

Strain	Percentage of growth rate reduction				
	NaCl	Sorbitol	H_2_O_2_	SDS	CR
P1 (wild-type)	65.3 ± 3.5%^A^	54.1 ± 1.0%^A^	80.2 ± 3.5%^A^	26.9 ± 2.7%^B^	40.5 ± 1.8%^A^
MS-2 (*Uvslt2*)	44.3 ± 6.7%^B^	16.5 ± 4.6%^B^	48.5 ± 10.7%^B^	43.3 ± 1.5%^A^	38.7 ± 0.7%^A^

*Growth rate was assayed with the wild-type strain P1 and *Uvslt2* mutant cultured on regular PSA or PSA with 0.6 NaCl, 0.6 M sorbitol, 0.05% H_2_O_2_, 0.2% SDS, or 300 μg/ml Congo red (CR). Mean and standard deviation were calculated with data from three replicates. For each strain, the growth rate on PSA with individual stresses was compared with that on regular PSA (arbitrarily set to 100%) to estimate the percentage of growth rate reduction. Differences in growth rate reduction between the wild-type and *Uvslt2* mutant were analyzed with the protected Fisher’s least significant difference (LSD) test. Different letters mark statistically significant differences (*P* ≤ 0.05).*

**FIGURE 6:**
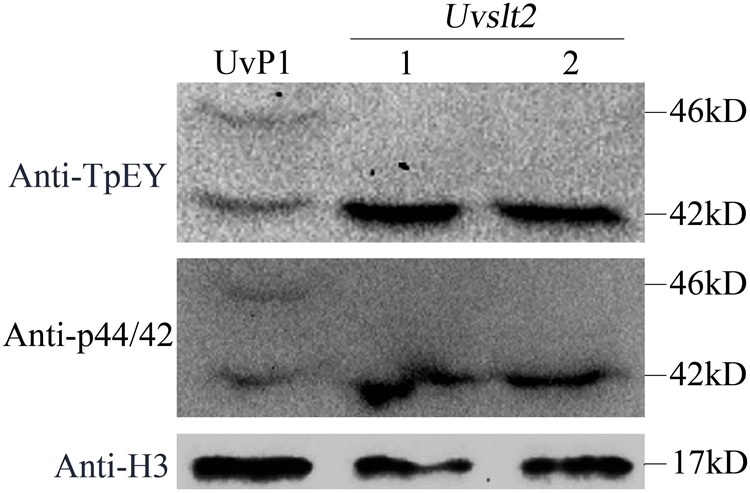
Assays for the phosphorylation of UvPmk1 and UvSlt2 MAP kinases. Western blots of total proteins isolated from the wild-type strain P1 and *Uvslt2* mutant were detected with an anti-TpEY phosphorylation specific antibody and an anti- p44/42 antibody. Phosphorylation and expression of UvSlt2 was detected in the wild-type but not in the *Uvslt2* mutant. Detection with an anti-H3 antibody (Cell Signaling Technology) was to show that similar amount of proteins were loaded for each sample.

Like in other filamentous fungi, UvSlt2 is one of the two MAP kinases with the TEY dual-phosphorylation motif in *U. virens*. Western blot analysis with the anti-TpEY antibody showed that *Uvslt2* deletion mutants had no detectable phosphorylation of UvSlt2 MAP kinase (**Figure 6**). However, phosphorylation of the other MAP kinase with the TEY motif that is orthologous to Pmk1 in the rice blast fungus *M. oryzae* (Li et al., 2017) was not affected (**Figure 6**). Similar amount of proteins from each sample was detected by anti-H3 antibody (**Figure 6**). These results further indicated the conserved nature of UvSlt2 as a MAP kinase in *U. virens*.

## Discussion

Targeted gene deletion and modification are important molecular tools to study gene functions in fungi and other organisms. In the budding yeast *Saccharomyces cerevisiae*, targeted gene deletion can be achieved by transforming a selectable marker flanked by homologous sequences as short as 20 bp (Ahn et al., 1988). In general, filamentous fungi have much lower homologous recombination frequency than *S. cerevisiae*. In *M. oryzae*, a model plant pathogenic fungus, the homologous gene replacement frequency is approximately 5% with 1-kb flanking sequences (Arazoe et al., 2015). For the rice false smut fungus *U. virens*, gene replacement was not effective by PEG-mediated transformation and occurred at <0.5% frequency by ATMT with 1-kb flanking sequences (Zheng et al., 2016). In this study, we showed that the efficiency of gene replacement by homologous recombination was significantly increased with the CRISPR-Cas9 system in *U. virens*. For the *USTA* gene, the gene replacement frequency was as high as 90% with gRNA spacer UA01. The significant increase in the gene replacement frequency by the CRISPR-Cas9 system will likely enable functional characterization of genes related to pathogenesis and mycotoxin production efficiently in *U. virens*, which is one of the major fungal pathogens of rice. In other filamentous fungi, the CRISPR-Cas9 system also increased the homologous gene replacement frequency (Arazoe et al., 2015; Liu et al., 2015). For example, the gene knockout frequency was increased from <5 to 25–53% in *Aspergillus fumigatus* with theCRISPR/Cas9 system (Fuller et al., 2015). Another major advantage of CRISPR is its capacity to introduce mutations to multiple genes with different gRNA (Liu et al., 2015). Because we used geneticin resistance to select for gene replacement mutants, the availability of useful selectable markers will be a limitation for applying this approach in *U. virens*. However, it remains possible that targeted deletion of two or more genes may be achieved by CRISPR with the same selectable marker in the rice false smut fungus.

In comparison with transforming the Cas9 and gRNA constructs sequentially into *U. virens*, the homologous gene replacement frequency was higher when they were transformed together on the same vector for all three *USTA* gRNA spacers. When spacer constructs were introduced into *U. virens* transformants expressing the Cas9 cassette, the homologous gene replacement frequency was 75, 60, and 60% for *USTA* gRNA spacers UA01, US13, and UA21, respectively. It was increased to 90, 86, and 87% when the Cas9 and gRNA spacers were transformed into *U. virens* on the same plasmid. In mammalian cells, similar observations have been reported because the Cas9 protein needs gRNA for the stabilization^20^. Among the three *USTA* spacers, UA01 had the highest predicted on-target score. It also had the highest gene replacement efficiency for deletion of *USTA* in *U. virens*. Therefore, it will be desirable to use gRNA spacers with the highest on-target score in other filamentous fungi for generating targeted gene knockout mutants by CRISPR-Cas9.

The U6 promoter is commonly used as the RNAP III promoter to control the expression of gRNA spacers in eukaryotes organisms (Gaj et al., 2013; Mefferd et al., 2015). However, the U6 promoter is not well-conserved in different fungi and it is approximately 500-bp in length. Furthermore, the gRNA spacers expressed from the U6 promoter have a G base at the 5′ end (Mefferd et al., 2015). Therefore, tRNA promoters also have been used as the RNAP III promoter to express gRNA spacers (Mefferd et al., 2015; Xie et al., 2015). In this study, we used the 72-bp Gln-tRNA promoter that has a typical cloverleaf structure^11,14^ to control the expression of gRNA spacers. Our results showed that the Gln-tRNA RNAP III promoter worked well to express gRNA spacers in *U. virens.* As a well-conserved promoter, it should also work in other plant pathogenic fungi for CRISPR-Cas9 studies.

One common concern is the off-target mutations of RGNs (Cho et al., 2014; Doench et al., 2016). However, none of the 20 *ustA* mutants assayed by sequencing analysis had mutations at seven predicted off-targets sites with 3–4 nucleotide variations for gRNA spacers UA13 and UA21 (UA13-3-1, UA13-4-2, UA13-4-3, UA21-3-1, and UA21-4-1). These results suggested that the mutation rate at off-targets with the CRISPR-Cas9 system is relatively low in *U. virens*. One possible explanation is that the *U. virens* genome is small (33.6 Mb) and not as complex as higher eukaryotic organisms (Zhang et al., 2014). In fact, all three *USTA* gRNA spacers have no predicted off-targets with only 1 or 2 nucleotide variations. The specificity of gRNA spacers is likely helpful to reduce mutations at off-targets. For the predicted off-targets of gRNA spacer UA01-1-1 and UA13-4-1, we failed to amplify the target sequences from the wild-type and *ustA* mutants with at least three different primer pairs tested for each. It is likely that the predicted off-target sequences of UA01-1-1 and UA13-4-1 in UV-8b, the strain used for genome sequencing^4^, may be different or absent in the wild-type strain P1 used in this study.

Unlike most small peptide mycotoxins synthesized by non-ribosomal peptide synthase (NRPS), ustiloxins are produced by post-translational modifications of UstA proteins synthesized on the ribosome. Although ustiloxins are present in the smut balls formed on infected rice kernels, to our knowledge, ustiloxin production have not been reported in *in vitro* cultures of *U. virens*. Under different culture conditions used to assay mycotoxin productions in *Fusarium* species, such as rice grain and cracked corn cultures (He et al., 2007; Jiang et al., 2016), we failed to detect ustiloxin production by the wild-type strain P1. Nevertheless, *USTA* is a single copy gene and encodes the only protein containing the ustiloxin peptide sequence in *U. virens*. Therefore, we expected that *ustA* deletion mutants will no longer produce ustiloxins. Unfortunately, because infection assays with *U. virens* is difficult and un-reliable, we failed to infect developing rice heads with the wild-type strain P1 and *ustA* mutants in repeated attempts. Thus, it remains to be determined whether ustiloxin production is important for virulence on rice in *U. virens*.

The Slt2 MAP kinase pathway is known to regulate cell wall integrity in *S. cerevisiae, M. oryzae*, and other fungi (Li et al., 2012; Jiang et al., 2018). The *Uvslt2* deletion mutant had increased sensitivities to cell wall and cytoplasmic membrane stresses in *U. virens*, which is consistent with its function in cell wall integrity. Interestingly, the *Uvslt2* mutant was more tolerant to hyperosmotic and oxidative stressors. In *U. virens*, UvHog1 is known to regulate responses to hyperosmotic and oxidative stresses (Zheng et al., 2016). It is possible that deletion of *UvSLT2* resulted in the hyper-activation of the UvHog1 pathway, which in turn increased the tolerance to hyperosmotic and oxidative stresses. Therefore, it will be important to characterize the crosstalk between these two MAP kinase pathways in *U. virens* in the future.

## Materials and Methods

### Strains, Plasmids, and Culture Conditions

All the *U. virens* strains were routinely cultured on YT (0.1% yeast extract, 0.1% tryptone, and 1% glucose) at 25°C. Conidiation was assayed with 6-day-old PSB (Potato Sucrose Broth) cultures. For ATMT (Zheng et al., 2016), *A. tumefaciens* strain AGL1 was used for the transformation of the wild-type strain P1 (Zheng et al., 2017), a field isolate provided by Dr. Yuanfeng Liu at Institute of Plant Protection, Jiangsu Academy of Agricultural Sciences. The binary T-DNA vector pDHt/sk-Ppdc-toCas9-Tpdc (pDHt/sk-PC) (Liu et al., 2015) was provided by Dr. Zhi-Hua Zhou at Shanghai Institutes for Biological Sciences, Chinese Academy of Sciences. Protoplast preparation and PEG-mediated transformation of *U. virens* strains were performed as described (Zheng et al., 2016). For transformation selection, hygromycin B (Calbiochem, La Jolla, CA, United States) and G418 (MP Biomedicals, Santa Ana, CA, United States) were added to the final concentration of 180 and 700 μg/ml, respectively, in the medium. The pCRISPR/Cas-U6-1 vector (Arazoe et al., 2015) was provided by Dr. Kuwata at Meiji University.

### Construction of the Cas9-gRNA Vectors With the *U. virens* Gln-tRNA Promoter

The Gln-tRNA-gRNA cassette containing two *Bsa*I sites was generated by fusion PCR with primers GRNA-F1, GRNA-R1, GRNA-F2, and GRNA-R2 (Supplementary Table S1). It was then cloned between the *Hin*dIII and *Eco*RI sites of vector pUC-H1-gRNA (Zheng et al., 2014) to generate the pUC19-tRp-gRNA plasmid. A short cassette with two *Bsm*BI sites was generated by annealing the sense (LINK-SEQ-F) and antisense (LINK-SEQ-R) oligonucleotides (Supplementary Table S1) and inserted between the two *Bsa*I sites of pUC19-tRp-gRNA by Golden Gate cloning (New England Biolabs, Ipswich, MA, United States) as described (Ran et al., 2013; Arazoe et al., 2015) to generate the pUC19- LINK vector. The P_Gln-tRNA_-gRNA cassette flanked by two *Bsm*BI sites was then released from pUC19-LINK and cloned between the *Kpn*I and *Eco*RI sites of pCRISPR/Cas-U6-1 (Arazoe et al., 2015) to generate the pCas9-tRp-gRNA vector. All the resulting plasmid vectors were verified by sequencing analysis.

### Construction of the Cas9-gRNA Vectors for Deletion of *USTA*

The gRNA spacers were designed with the gRNA designer program^1^ for best on-target scores (Doench et al., 2014, 2016) and then analyzed with the Cas9off program^2^ to identify potential off-targets (Guo et al., 2014). Three *USTA* gRNA spacers, UA01, UA13, and UA21, were selected by weighing both on-target scores and potential off-targets. The sense and antisense oligonucleotides of each gRNA spacer (Supplementary Table S1) were synthesized and annealed to generate corresponding gRNA spacers as described (Arazoe et al., 2015). The resulting gRNA spacers were cloned between the two *Bsa*I sites of pUC19-tRp-gRNA or the two *Bsm*BI sites of pCas9-tRp-gRNA by Golden Gate cloning (New England Biolabs).

### Generation of the *USTA* Gene Replacement Constructs and Mutants

The 1.01-kb upstream and 1.09-kb downstream flanking sequences of *USTA* were amplified with primer pairs of USTA1F/USTA2R and USTA3F/USTA4R (Supplementary Table S1), respectively, and fused with the geneticin-resistance (GenR) cassette from pFL2 (Zhou et al., 2011) by double-joint PCR. For sequential transformation, the resulting PCR products were cloned into pUC19-tRp-gRNA and transformed into protoplasts of transformant CS-2 expressing the pDHt/sk-PC Cas9 construct as described (Zheng et al., 2016). The same *USTA* knockout PCR products also were cloned into the pCas9-tRp-UA01, pCas9-tRp-UA13, and pCas9-tRp-UA21 vectors and then transformed into protoplasts of the wild-type strain P1. G418-resistant transformants were screened for deletion of *USTA* by PCR with primers USTA/5F and USTA/6R, and further verified by PCR with primer pairs USTA7F/G855R and USTA8R/G856F (Supplementary Table S1).

To delete *USTA* by the conventional gene replacement approach, its upstream and downstream flanking sequences were amplified as described above and fused with the hygromycin phosphotransferase gene (*hph*) from pCB1003 (Carroll et al., 1994) by overlapping PCR as described (Zheng et al., 2016). The resulting PCR products were cloned into the binary vector pCBDW and transformed into stain P1 by ATMT as described (Zheng et al., 2016). Hygromycin-resistant transformants were screened for *ustA* deletion mutants by PCR.

### Analysis of Off-Target Mutations by CRISPR-Cas9

The potential off-target sites of *USTA* spacers UA01, UA13, and UA21 with 1 to 5 nucleotide variations were predicted with the Cas9off program (Guo et al., 2014). For each gRNA spacer, the predicted off-targets with less than four nucleotide differences were amplified from 10 corresponding *ustA* deletion mutants and sequenced for possible mutations.

### Generation of the *UvSLT2* Gene Replacement Construct and Mutants

For *UvSLT2*, the gRNA spacer SLT01 was selected for its highest on-target score and generated by annealing the sense and antisense oligonucleotides (Supplementary Table S1). The resulting products were cloned between the two *Bsm*BI sites of pCas9-tRp-gRNA by Golden Gate cloning to generate pHY2016A (**Table 1**). The 1.25-kb upstream and 1.04-kb downstream flanking sequences of *UvSLT2* were amplified and fused to the Gen^R^ cassette by double-joint PCR (Hou et al., 2002). The resulting PCR products were cloned into pHY2016A and transformed into protoplasts of strain P1. To generate *UvSTL2* deletion mutants by ATMT, its upstream and downstream flanking sequences were fused with the *hph* cassette from pCB1003 (Carroll et al., 1994) by overlapping PCR and cloned into pCBDW for transformation of strain P1 as described (Zheng et al., 2016). The *Uvslt2* deletion mutants were screened and verified by PCR with primers listed in Supplementary Table S1.

### Western Blot Analysis for Assaying the Phosphorylation of UvSlt2

Total proteins were isolated from vegetative hyphae as described (Hou et al., 2015). For western blot analysis, total proteins (20 μg) were separated on a 10% SDS-PAGE gel and transferred to the nitrocellulose membrane. Phosphorylation of the UvPmk1 and UvMps1 MAP kinases was detected with the PhophoPlus p44/42 MAP kinase antibody (Cell Signaling Technology, Danvers, MA, United States) as described (Liu et al., 2011). Detection with an anti-p44/42 antibody (Cell Signaling Technology, Danvers, MA, United States) was to show the expression levels of Slt2 in these samples.

## Author Contributions

YL and J-RX conceived the experiments. YL, YH, and CJ conducted the experiments and analyzed data. YL, CJ, CW, and J-RX prepared and revised this manuscript.

## Conflict of Interest Statement

The authors declare that the research was conducted in the absence of any commercial or financial relationships that could be construed as a potential conflict of interest.

## Footnotes

1 portals.broadinstitute.org/gpp/public/analysis-tools/sgrna-design

2 github.com/wangqinhu/cas9off
