# Supplementary material for: Targeted Deletion of the USTA and UvSLT2 Genes Efficiently in Ustilaginoidea virens With the CRISPR-Cas9 System
Source: Front Plant Sci. 2018 May 24;9:699. doi: 10.3389/fpls.2018.00699 (PMC5976777; doi:10.3389/fpls.2018.00699)
Supplement: Supplementary file 1 [file Presentation_1.PDF]

Supplementary Material

Targeted deletion of the *USTA* and *UvSLT2* genes efficiently in *Ustilaginoidea virens* with the CRISPR-Cas9 system

Yafeng Liang , Yu Han , Chenfang Wang , Cong Jiang , Jin-Rong Xu*

*** Correspondence:** Corresponding Author: jinrong@purdue.edu

1 Supplementary Data

2 Supplementary Figures and Tables

For more information on Supplementary Material and for details on the different file types accepted, please see here.

2.1 Supplementary Figures

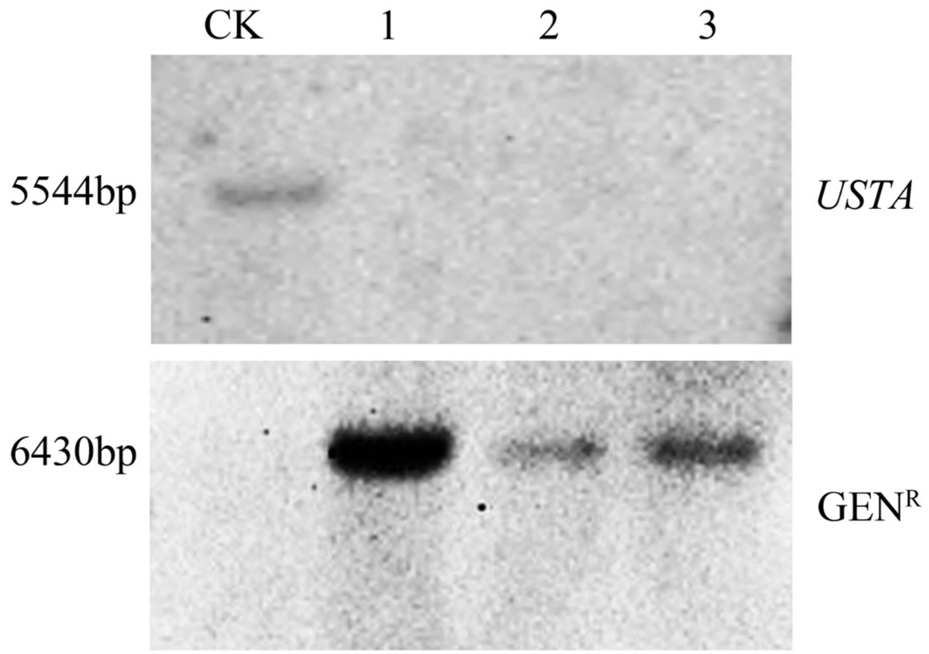

Supplementary Figure 1. Southern blot analysis of the *UvustA* deletion mutants. Southern blots of *Hin*dIII-digested DNA isolated from the wild-type strain P1 (CK) and three *ustA* mutants

generated with pCas9-tRp-gRNA-UA01 were hybridized with an *USTA* fragment (upper panel) and a fragment of the geneticin-resistance (GEN) marker (lower panel) as the probes. The 5.5-kb *USTA* band was detected only in strain P1 but the 6.4-kb GEN band were detected in all three ustA deletion mutants.

2.2 Supplementary Tables

Table S1. Primers used in this study

NAME	SEQUENCE (5′ TO 3′)	EXPERIMENT
GRNA-F0	CCCAAGCTTGGTTCCATGGTCTAACGGTTATGACTG	Cloning of *tRp-gRNA*
GRNA-F1	GGTTCCATGGTCTAACGGTTATGACTGCGGATTCTGATTCCGCCAGCGAGGGT TCGACTCCCCCTGGAACCTCGAGACCTCGGTACCAGGGTCTCGG	Gln-tRNA-gRNA fusion PCR primer
GRNA-R1	GCTATTTCTAGCTCTAAAACCGAGACCCTGGTACCGAGGTCTCG	Gln-tRNA-gRNA fusion PCR primer
GRNA-F2	GGTTTTAGAGCTAGAAATAGCAAGTTAAAATAAGGCTAGTCCGTTATCAACT TGAAAAA	Gln-tRNA-gRNA fusion PCR primer
GRNA-R2	AAAAGCACCGACTCGGTGCCACTTTTTCAAGTTGATAACGGACTAGC	Gln-tRNA-gRNA fusion PCR primer
GRNA-R0	CCGGAATTCAAAAAAAAGCACCGACTCGGTGC	Cloning of *tRp-gRNA*
C9TSG-F	CGGGGTACCGGTTCCATGGTCTAACGGTTATGA	Cloning of pCAS9-tRp-gRNA
C9TSG-R	CCGGAATTCAAAAAAAAGCACCGACTC	Cloning of pCAS9-tRp-gRNA
LINK-SEQ-F	ACCTCGAGACGTCGGATCCAGCGTCTCG	Cloning ofpUC-LINK
LINK-SEQ-R	AAACCGAGACGCTGGATCCGACGTCTCG	Cloning of pUC-LINK
UA01-F	ACCTAATGGCGTAGTCCTCAACAG	gRNAUA01 primers
UA01-R	AAACCTGTTGAGGACTACGCCATT	gRNA UA01 primers
UA13-F	ACCTCACTGCAGCAGGAAACCCAG	gRNAUA13 primers
UA13-R	AAACCTGGGTTTCCTGCTGCAGTG	gRNAUA13 primers
UA21-F	ACCTTCATCTCCATCTTGGCCACT	gRNAUA21 primers
UA21-R	AAACAGTGGCCAAGATGGAGATGA	gRNAUA21 primers

SLT2-01-F	ACCTCGGTAAATGGTCAGACGAAC	gRNASLT01 primers
SLT2-01-R	AAACGTTCGTCTGACCATTTACCG	gRNA SLT01 primers
UstA-g1-4-F	ACAACAGCTTCCAGTTGATCC	Off-target primers
UstA-g1-4-R	CCCGAGACCATCGACAAGC	Off-target primers
UstA-g13-3-F	GTGGCAGAGCTCCAAGCAT	Off-target primers
UstA-g13-3-R	TGCCAAGCGAATACAATTCCAG	Off-target primers
UstA-g13-4-1-F	CTAATGTCCGGGACGTGCAT	Off-target primers
UstA-g13-4-1-R	GTCTCCAACGCAGGAAGACA	Off-target primers
UstA-g13-4-2-F	GGTTCCGGCCAGTTAAAAGC	Off-target primers
UstA-g13-4-2-R	GGAACCAGAATTGCTTGGCG	Off-target primers
UstA-g13-4-3-F	GGGACCACACCAAGGTGATT	Off-target primers
UstA-g13-4-3-R	GTGCATCCAAGAGGTAGCGT	Off-target primers
UstA-g21-3-F	ACGATTTCTCGCATCACCCG	Off-target primers
UstA-g21-3-R	CCCATGAGTCTGAGTCGCTG	Off-target primers
UstA-g21-4-F	CGCATACCGCGCAGAAAATA	Off-target primers
UstA-g21-4-R	TCGGGGACGATGACGGTAT	Off-target primers
UstA-g1-4-F3	GTGCGTTTCGAAGATGCTGC	Off-target primers
UstA-g13-4-1-F3	GTCGCTCGAGCTTCCCATAG	Off-target primers
UstA-g13-4-1-R3	ACCAAAGGGCAGTTCTCCTG	Off-target primers
GEN/F	GAGGTTGCGATTTCTCTGCCGTATCTG	Amplification of G418
GEN/R	GCCAGCAGTAGACACTTGGAATCTAAAC	Amplification of G418
G852/F	TCGGCTATGACTGGGCACAACA	Amplification of G418
G850/R	GAGCGGCGATACCGTAAAGCAC	Amplification of G418
G855/R	TGTTGGGTTTGAGCTAGGTGGG	Gene replacement mutant screening
G856/F	GAATGGTCAAATCAAACTGCTAGATAT	Gene replacement mutant screening

USTA/1F	CCGGTAAGCCGACAACAAGA	Amplifying the 5' flanking region of *ustA*
USTA/2R	CAGATACGGCAGAGAAATCGCAACCTCTGCAATTAACCCGTGTGAGGT	Amplifying the 5' flanking region of *ustA*
USTA/3F	GTTTAGATTCCAAGTGTCTACTGCTGGCCCATGACAGAGAGGAACCCG	Amplifying the 3' flanking region of *ustA*
USTA/4R	GAGTCGAGACCTTGGCGAAA	Amplifying the 3' flanking region of *ustA*
USTA/5F	GTTTCCTGCTGCAGTGGTTG	Screening for *ustA* deletion mutants
USTA/6R	TGCCATGCAGCTGTTAATTCC	Screening for *ustA* deletion mutants
USTA/7F	TGGCGACTGACATGATCTGC	Screening for *ustA* deletion mutants
USTA/8R	GGAGCCTCGCAAGTACAACA	Screening for *ustA* deletion mutants
SLT2/1F	CACAGGTTACGCCCACTCCG	Amplifying the 5' flanking region of *UvSLT2*
SLT2/2R	CAGATACGGCAGAGAAATCGCAACCTCACAGCAGGGGACCGTAGAGT	Amplifying the 5' flanking region of *UvSLT2*
SLT2/3F	GTTTAGATTCCAAGTGTCTACTGCTGGCGAGGCTGGGAAACACGGGGAT	Amplifying the 3' flanking region of *UvSLT2*
SLT2/4R	CGTCTATCGGGTCCTGACTGCC	Amplifying the 3' flanking region of *UvSLT2*
SLT2/5F	GCGTTGCCATCAAGAAAGTCAC	Screening for *Uvslt2* deletion mutants
SLT2/6R	CGCAAAGAATCTGGTAAATAAACGACT	Screening for *Uvslt2* deletion mutants
SLT2/7F	GACGAGTAAACGCAGAGGAAG	Screening for *Uvslt2* deletion mutants
SLT2/8R	TGCTCTACATAGCCCTCGGT	Screening for *Uvslt2* deletion mutants
tCAS9-F1	AGTACAGCATTGGCCTGGAC	Verifying the CAS9
tCAS9-R1	CGATCAGGTTCTCGAGTCGG	Verifying the CAS9
